# Effects of prenatal depressive symptoms on maternal and infant cortisol reactivity

**DOI:** 10.1007/s00737-016-0611-y

**Published:** 2016-03-04

**Authors:** Elizabeth C. Braithwaite, Susannah E. Murphy, Paul G. Ramchandani

**Affiliations:** 1 Department of Experimental Psychology, University of Oxford, 9 South Parks Road, Oxford, OX1 3UD UK; 2 Department of Psychiatry, University of Oxford, Warneford Hospital, Oxford, OX3 7JX UK; 3 Centre for Mental Health, Imperial College London, 7th Floor Commonwealth Building, Du Cane Road, London, W12 0NN UK

**Keywords:** Prenatal depression, Development, Cortisol, HPA axis

## Abstract

Prenatal depression is associated with adverse offspring outcomes, and the prevailing mechanistic theory to account for mood-associated effects implicates alterations of the maternal and foetal hypothalamic-pituitary adrenal (HPA) axes. Recent research suggests that depression may be associated with a failure to attenuate cortisol reactivity during early pregnancy. The aim of the current study is to investigate whether this effect continues into mid and late gestation. A further aim is to test whether maternal prenatal cortisol reactivity directly predicts infant cortisol reactivity. One hundred three pregnant women were recruited during either the second or third trimester. Depressive symptoms were assessed by self-report, and maternal salivary cortisol responses to a stressor (infant distress film) were measured. Approximately 2 months after birth, mothers (*n* = 88) reported postnatal depression and infant salivary cortisol responses to inoculation were measured. Prenatal depression was not associated with cortisol reactivity to acute stress in mid and late pregnancy. Similarly, neither prenatal depression nor maternal prenatal cortisol reactivity predicted infant cortisol reactivity to inoculation at 2 months. If the effects of prenatal depression on foetal and infant development are mediated by alterations of the maternal and foetal HPA axes, then early pregnancy may be a particularly vulnerable period. Alternatively, changes to HPA reactivity may not be as central to this association as previously thought.

## Introduction

Accumulating evidence suggests that symptoms of prenatal psychological distress (encompassing feelings of depression, anxiety and stress) increase risk for a number of adverse offspring outcomes. For example, prenatally distressed women are at increased risk for both preterm birth (Class et al. 2011; Copper et al. 1996; Nkansah-Amankra et al. 2010) and of having a low birth weight baby (Sable and Wilkinson 2000; Zhu et al. 2010). Exposure to maternal prenatal distress has also been linked with increased rates of behavioural difficulties in childhood (O’Connor et al. 2002; O’Connor et al. 2003), and psychiatric disorders in adolescence (Pearson et al. 2013; Van den Bergh et al. 2008a). Interestingly, such effects may be independent of shared risk genes between mother and infant (Rice et al. 2010), and also independent of maternal postnatal psychological distress (O’Connor et al. 2003; Pearson et al. 2013). Thus, it is possible that in utero biological mechanism(s) mediate, at least in part, the link between exposure to maternal prenatal psychological distress and adverse offspring outcomes.

The prevailing mechanistic theory in the field of perinatal psychiatry to account for mood-associated effects on offspring implicates alterations of the maternal and foetal hypothalamic-pituitary adrenal (HPA) axes (Braithwaite et al. 2014; Glover 2011; Talge et al. 2007). It has been suggested that mood-associated increases in maternal glucocorticoids induce a decreased expression of the placental enzyme 11B-HSD2 (O’Donnell et al. 2012), resulting in more active transfer of cortisol into foetal circulation. Such increases in foetal cortisol may perturb the development of the foetal HPA axis. Indeed, supporting evidence suggests that infants, children and adolescents born to prenatally depressed mothers show exaggerated cortisol responses to acute stress (Brennan et al. 2008; Davis et al. 2011b; O’Connor et al. 2005; Van den Bergh et al. 2008b), and such increased cortisol reactivity has been related to symptoms of depression in adolescence (Van den Bergh et al. 2008b). However, there are flaws in the HPA programming model and the supporting evidence. For example, almost no studies have demonstrated a mediating role of maternal cortisol in the association between prenatal depression and offspring outcomes, and often only maternal depression or cortisol is shown to be independently associated with offspring behaviour and HPA function (Davis and Sandman 2010; Gutteling et al. 2004; Sarkar et al. 2008).

Furthermore, while there is accumulating evidence in support of an altered offspring HPA axis following exposure to prenatal mood disturbance, the link between prenatal mood disturbance and raised maternal glucocorticoids is less clear. In non-pregnant populations, symptoms of depression are associated with cortisol hyper-secretion (Bhagwagar et al. 2005; Cowen 2002; Herbert 2013). However, attempts at characterising depression-associated hyper-cortisol release in pregnancy have provided mixed results, with evidence both for (Giesbrecht et al. 2012; Murphy et al. 2015; O’Connor et al. 2013; Obel et al. 2005) and against (Evans et al. 2008; Hellgren et al. 2013; Pluess et al. 2012) cortisol hyper-secretion in women experiencing symptoms of depression. A likely explanation for the disparate findings is that cortisol levels rise throughout pregnancy, regardless of mood state, due to the release of corticotrophin-releasing hormone (CRH) from the placenta. By term, cortisol levels are higher than in the non-pregnancy state (Lindsay and Nieman 2005), and therefore detecting mood-associated changes in cortisol becomes difficult.

An alternative explanation is that existing studies have typically measured diurnal cortisol release during pregnancy. However, recent research from our group suggests that assessments of HPA *reactivity* to stimulation may be a more effective method for detecting mood-associated cortisol hyper-secretion. In non-depressed pregnant populations, it has been well documented that cortisol reactivity to acute stress attenuates during gestation (De Weerth et al. 2007; Entringer et al. 2010; Nierop et al. 2006), which may be an adaptive process in order to protect the foetus from fluctuating glucocorticoid levels (de Weerth and Buitelaar 2005b). In a previous study, we exposed 53 participants to an infant distress stimulus during early pregnancy, and recorded psychological and cortisol responses. Although all participants reported increases in state anxiety in response to the stimulus, only those with symptoms of depression showed a significant increase in salivary cortisol (Murphy et al. 2015). Thus, we suggest that prenatal depression may be associated with a failure of the usual attenuation in cortisol reactivity.

However, as our previous study was carried out during early pregnancy, a critical outstanding question is whether this effect may persist into later gestation, when circulating cortisol levels are higher regardless of mood state. The primary aim of this study is to investigate the effects of depression on cortisol reactivity in mid and late pregnancy, and we hypothesise that depressive symptoms will continue to be associated with a failure to attenuate cortisol reactivity throughout gestation. It is also unclear whether maternal cortisol reactivity may directly predict infant cortisol reactivity, as would be expected given the mechanistic model of maternal and foetal HPA programming. Thus, the secondary aim of this study is to directly test this theory, and we hypothesise that maternal cortisol reactivity will strongly predict infant cortisol reactivity.

## Methods

### Participants

One hundred three pregnant women were recruited to this study during either the second or third trimester of pregnancy. All participants were primiparous, more than 14 weeks pregnant, had a singleton pregnancy, were over the age of 18, had no medical complications associated with their pregnancy and were not currently taking steroid-based medications. This research study was reviewed and approved by the Research Ethics Committee South Central Oxford B (REF: 12/SC/0473), and all participants provided informed consent.

### Procedure

#### Prenatal assessment

Participants were invited to a prenatal test session, which took place either at the Department of Psychiatry, Warneford Hospital, or at the participant’s home. These sessions all took place between the hours of 1 pm and 7 pm, and lasted approximately 90 min. Participants were asked to complete a questionnaire, which contained questions about their demographic characteristics and current levels of mood. Participants were then asked to watch a short film depicting distressed young infants, all under the age of 6 months. The film was 6 min in length and included eight short clips of crying infants. The clips were taken from online sources with permission from the owners. This video has been used in a previous study (Murphy et al. 2015) and found to induce significant salivary cortisol responses in a group of late first/early second trimester pregnant women with symptoms of depression. During the film, participants were asked to wear headphones, sit quietly and watch the film. Saliva samples were collected at five time points during the test session using saliva collection aids and plastic cryovials (Salimetrics, UK). Two samples were taken before the film, approximately 20 min apart. The third sample was taken immediately after the film, and the fourth and fifth samples were taken 10 and 20 min after the film, respectively. Saliva samples were stored at −20 °C until analysis. Before and after the film, participants rated their mood and also their psychological response to the film on three visual analogue scales.

#### Postnatal assessment

All participants were invited to take part in a postnatal follow up, of which 88 agreed. These participants were visited at home approximately 2 months after they had given birth, and reported postnatal mood symptoms. Mothers were asked to collect four saliva samples from their infant on the day of their first inoculation at 8 weeks of age. Of the 88 participants who took part in the postnatal study, 74 obtained and returned infant saliva samples. Sixty participants collected samples at the 8-week appointment. However, in some cases, it was not possible for mothers to collect infant saliva at this appointment, and instead collected saliva from their infants at their 12-week (*n* = 10) or 16-week (*n* = 4) inoculations. On average, infants were 68 days old (range = 50 to 177 days) when they received inoculations and saliva samples were collected. Samples were collected using Infant Saliva Swabs and Swab Storage Tubes (Salimetrics, UK). Mothers collected the first sample any time during the day before the inoculation, the second sample immediately after inoculation, and the third and fourth samples 20 and 40 min following inoculation respectfully. Participants were provided with four Infant Saliva Swabs and four Swab Storage Tubes, and a stamped-addressed envelope to return the samples. The samples were shipped at room temperature, and would have remained at room temperature for no longer than 24 h. These samples were then centrifuged on receipt, and stored at −20 °C until analysis. Participants were also asked to record the time of sample collection, the time of inoculation, the time of the infants last feed before inoculation and whether that feed was breast milk or formula.

### Measures

#### Maternal symptoms of depression

Maternal pre- and postnatal depressive symptoms were self-reported using the Edinburgh Postnatal Depression Scale (EPDS). The EPDS is the most widely used self-report questionnaire to identify symptoms of depression during the perinatal period. The scale consists of 10 items that describe common symptoms of depression, each item is scored from 0 to 3, and the scale has a maximum score of 30. A score of 13 or above is indicative of clinical levels of depression; however, for research purposes, a cut off score of 10 is frequently used to identify a group ‘at risk’ of depression (Adewuya et al. 2006; Adouard et al. 2005; Bergink et al. 2011; Felice et al. 2004; Murray and Cox 1990). A recent study has shown that using a cut off of 10 in the second and third trimester of pregnancy provides a good balance between sensitivity (70–79 %) and specificity (96–97 %) (Bergink et al. 2011), and we have used this cut off in a previous study (Murphy et al. 2015). Thus, in the current study, participants who scored 10 or above on the EPDS comprised the ‘depression-symptom’ group, whereas participants who scored 9 and below were the control group.

### Maternal psychological responses to infant distress stimulus

#### Spielberger State Anxiety Inventory (SSAI)

This is a 20-item questionnaire used to identify symptoms of state anxiety, such as ‘I feel calm’, ‘I feel tense’ and ‘I am worried’. Participants were required to rate their responses on a 4-point scale from ‘not at all’ to ‘very much’. The maximum score on this questionnaire is 60, and higher scores indicate higher levels of state anxiety. The SSAI has shown good internal consistency (0.86–0.95) and test re-test reliability (0.65–0.75) (Spielberger et al. 1983). This measure was administered both pre- and post-film.

### Visual analogue scales

After the film, participants were also asked to complete three visual analogue scales, rating ‘how much did you want to comfort the baby?’, ‘how upsetting did you find the film?’ and ‘how good do you think you would be at comforting the baby?’.

### Salivary cortisol

Salivary cortisol concentrations were quantified using an enzyme immunoassay kit, sourced from Salimetrics UK, and analysis was carried out in accordance with the manufacturer’s instructions. Samples were analysed in singlets, and the minimum detectable concentration was 0.2 nmol/l when a 0.1-ml volume was assayed. Cortisol outliers that were more than three standard deviations from the mean were excluded (20 of 1055 data points excluded).

### Statistical analysis

For analysis of the prenatal data, participants were divided into two groups based on their EPDS score: those who scored 9 or below comprised the control group (*n* = 79) and those who scored 10 or above comprised the depressive-symptom group (*n* = 24). The demographic characteristics of the two groups were compared using *t* test and chi-squared tests. Pearson’s bivariate correlations were used to assess associations between demographic variables, and salivary cortisol measures. Repeated measures ANOVAs were used to assess changes in mood and salivary cortisol in response to the infant distress stimulus. Time was used as a within-subjects factor, and group (depressive-symptom vs. control) and trimester (2nd vs. 3rd) as between-subjects factors.

For analysis of the postnatal data, characteristics of the control infants (*n* = 67) and depression-exposed infants (*n* = 21) were compared using *t* tests and chi-squared tests. Correlations between infant characteristics, maternal mood and infant cortisol were then assessed using Pearson’s bivariate correlations. For consistency with the antenatal data, the infant cortisol data was analysed using a repeated measured ANOVA in order to assess changes in cortisol concentration over time. Time was entered as a within-subjects factor, and group (depression-exposed vs. control) and infant gender were entered as between-subjects factors. Maternal postnatal depression was used as a covariate in this analysis, as was maternal trimester at antenatal assessment and infant age at the time of inoculation. This data was re-analysed using linear regression models to assess whether maternal prenatal cortisol reactivity (to infant distress stimulus) directly predicted infant cortisol reactivity (to inoculation).

## Results

### Prenatal data

#### Sample characteristics

Demographic characteristics are presented in Table 1. This primarily Caucasian sample of women was highly educated, had a mean age of 31 and all participants were primiparous. At the time of assessment, the mean gestational length was 190.4 days (range = 104–281 days). Fifty (48.5 %) women were in their second trimester (gestational range = 104–188 days) and 53 (51.5 %) were in their third trimester (gestational range = 190–281 days). The demographic variables of the control and depressive-symptom groups were mainly comparable, but differed in ethnicity; the control group contained a higher proportion of Caucasian participants than the depressive-symptom group (*X*^2^_(6)_ = 15.750, *p* = 0.015). As expected, the groups also differed in prenatal depression score, with the depressive-symptom group scoring significantly higher than the control group (*T*_(101)_ = −13.286, *p* < 0.001). There were no significant correlations between any of the demographic characteristics. Gestation was significantly correlated with the maternal salivary cortisol measures, reflecting the expected increase in salivary cortisol concentrations across gestation; however, gestation was not related to the degree of cortisol change in response to the stressor.

**Table 1:** Demographic characteristics of the sample

Demographic variables	Control group (*n* = 79)	Depressive-symptom group (*n* = 24)
Age (m, SD)	31.42 (4.72)	31.67 (4.42)
Education (*n*, %)		
GCSE/O-level	1 (2.5)	–
A-level	2 (2.5)	1 (4.2)
Undergraduate degree	29 (36.7)	4 (29.2)
NVQ	4 (5.1)	3 (12.5)
Postgraduate degree	43 (54.4)	13 (54.2)
Ethnicity (*n*, %)		
Caucasian	76 (96.2)	18 (75)
Black	–	1 (4.2)
Asian	2 (2.5)	3 (12.5)
Chinese	–	2 (8.3)
Mixed Race	1 (1.3)	–
Alcohol units/week (*n*, %)		
None	67 (84.8)	19 (79.2)
1–5	12 (15.2)	5 (20.8)
Cigarettes/week (*n*, %)		
None	65 (82.3)	21 (87.5)
Did not respond	14 (17.7)	3 (12.5)
Trimester (*n*, %)		
Second	41 (51.9)	9 (37.5)
Third	38 (48.1)	14 (62.5)
Planned pregnancy (*n*, %)	67 (84.8)	21 (87.5)
Previous history of mental health problems (*n*, %)	17 (21.5)	12 (50)
Prenatal depression (m, SD)	3.95 (2.99)	13.58 (3.49)

Prenatal depression was assessed using the Edinburgh Postnatal Depression Scale

*GCSE* general certificate in secondary education, *NVQ* national vocational qualification

#### Psychological responses to the infant distress stimulus

At baseline, participants in the depressive-symptom group had significantly higher scores on state anxiety scale (*F*_(1)_ = 32.13, *p* < 0.001). A repeated measures ANOVA was used to assess changes in mood scores from pre- to post-film. There was a significant main effect of time, reflecting an increase in state anxiety following film viewing in both groups (*F*_(1)_ = 65.45, *p* < 0.001), and a main effect of group (*F*_(1)_ = 36.27, *p* < 0.001), reflecting the relatively increased state anxiety scores at both time points in the depression-symptom group. However, there was no significant interaction between time and group (*F*_(1)_ = 1.63, *p* = 0.205), suggesting that the two groups did not differ in the magnitude of change in state anxiety from pre- to post-film, see Table 2. On the three visual analogue scales, there were no significant differences between the groups on how they rated their desire to comfort the infants (*F*_(1)_ = 0.142, *p* = 0.288), how upsetting they found the film (*F*_(1)_ = 0.350, *p* = 0.555) or how good they thought they would be at comforting the infants (*F*_(1)_ = 0.802, *p* = 0.373).

**Table 2:** Psychological responses to the infant distress film

	Control group (*n* = 79)		Depressive-symptom group (*n* = 24)	
	Pre-film	Post-film	Pre-film	Post-film
State anxiety (mean, SD)	24.94 (4.78)	32.57 (9.80)	33.96 (10.08)	44.50 (10.47)
VAS “How much did you want to comfort the baby?” (mean, SD)		8.01 (2.27)		7.40 (2.32)
VAS “How upsetting did you find the film?” (mean, SD)		5.16 (2.34)		4.98 (2.40)
VAS “How good do you think you would be at comforting the baby?” (mean, SD)		6.66 (1.72)		6.03 (2.71)

*VAS* visual analogue scale

#### Cortisol reactivity to the infant distress stimulus

**Fig. 1:**
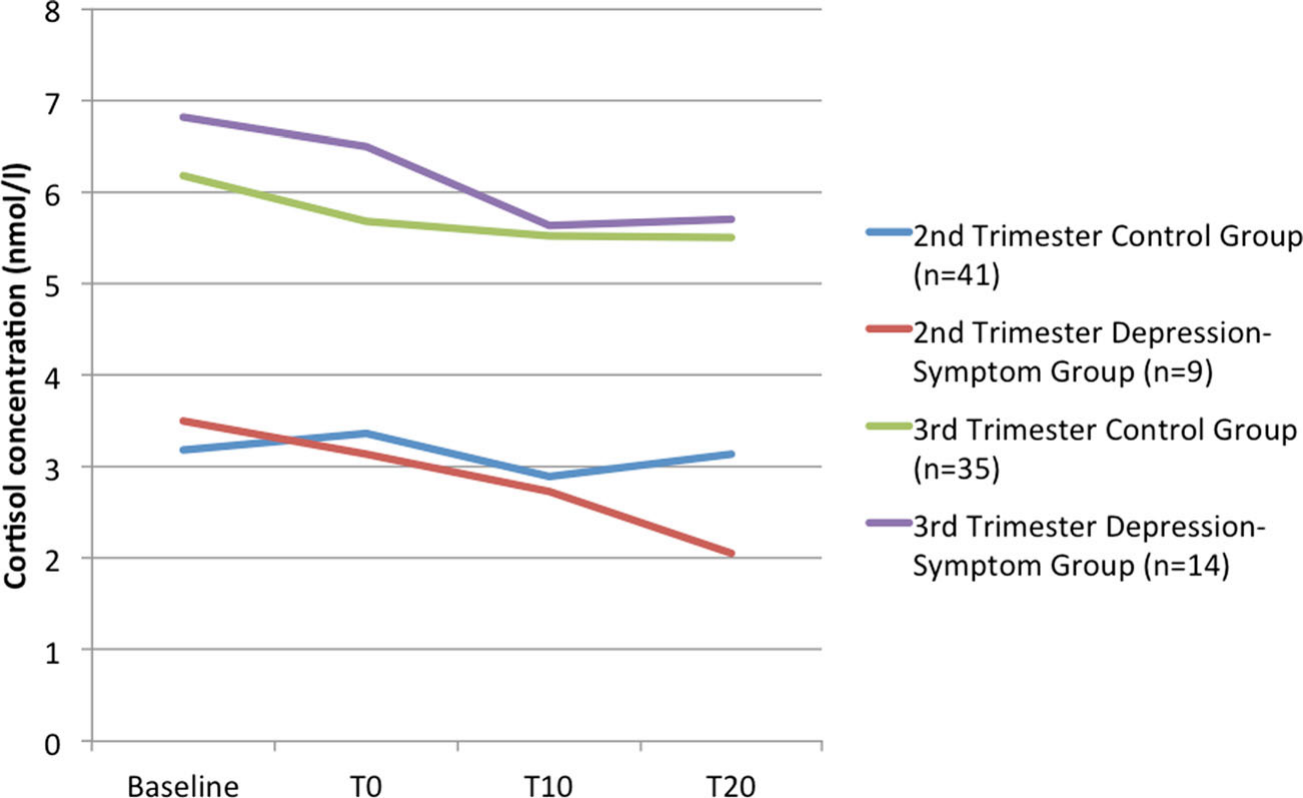
Maternal salivary cortisol responses to the infant distress film, split by group and trimester

A repeated measures ANOVA was used to assess change in cortisol concentrations over the test session. There was no within-subjects effect of time (*F*_(3)_ = 0.697, *p* = 0.557), which reflects no change in cortisol concentrations in response to the film. Similarly, there were no interactions between time, trimester and group, suggesting that participants from both groups and both the second and third trimester did not show a change in salivary cortisol across the test session. There was a main effect of time of day on cortisol concentrations (*F*_(1)_ = 4.288, *p* = 0.050); the negative correlation between time of day of assessment and baseline cortisol (*r* = −0.543, *p* < 0.001) reflects the diurnal cortisol decline. There was also a significant between-subjects effect of trimester on salivary cortisol (*F*_(1)_ = 4.305, *p* = 0.049), akin to higher cortisol concentrations in the third trimester participants. However, there were no between-subjects effects of group (*F*_(1)_ = 0.646, *p* = 0.430), or a group × trimester interaction (*F*_(1)_ = 3.724, *p* = 0.203). This is demonstrated graphically in Fig. 1. When location of the prenatal assessment (Department of Psychiatry vs. participant’s home) was included as a covariate in the analyses, there was no change to the results.

### Postnatal data

#### Demographic characteristics

Of the 88 infants included in this study, 67 were born to mothers of the control group, and 21 were born to mothers of the depressive-symptom group. The two groups were comparable on all birth outcomes apart from gestational age at birth; those infants born to depressed mothers had slightly older gestational ages at delivery than those born to control mothers (*t*_(85)_ = −1.995, *p* = 0.049). Demographic characteristics of the infants are presented in Table 3.

**Table 3:** Infant demographic characteristics

Demographic variables	Control infants (*n* = 67)	Depression-exposed infants (*n* = 21)
Infant characteristics		
Gender (*n*, %)		
Male	28 (42.2)	11 (52.4)
Female	39 (57.8)	10 (47.6)
Birth weight, Kg (m, SD)	3.84 (0.46)	3.33 (0.90)
Gestational age at birth, weeks (m, SD)	40 (1.14)	40.6 (1.12)
Delivery method (*n*, %)		
Vaginal	35 (52.2)	9 (42.9)
Forceps	16 (23.9)	4 (19)
Ventouse	6 (9)	3 (14.3)
Elective caesarean	2 (3)	1 (4.8)
Emergency caesarean	8 (11.9)	4 (19)
Delivery complications (*n*, %)	28 (42.4)	7 (33.3)
Age at postnatal assessment, weeks (m, SD)	8.4 (1.78)	9.2 (1.92)
Infant behaviour (m, SD)		
Activity	3.44 (0.67)	3.65 (0.79)
Smiling	4.13 (1.50)	4.11 (1.06)
Distress	4.20 (0.75)	3.99 (0.78)
Soothing	4.41 (1.00)	4.23 (0.90)
Maternal mood		
Prenatal depression (m, SD)	4.0 (2.91)	13.43 (3.49)
Postnatal depression (m, SD)	6.68 (3.63)	8.38 (4.99)

#### Infant cortisol reactivity to inoculation

**Fig. 2:**
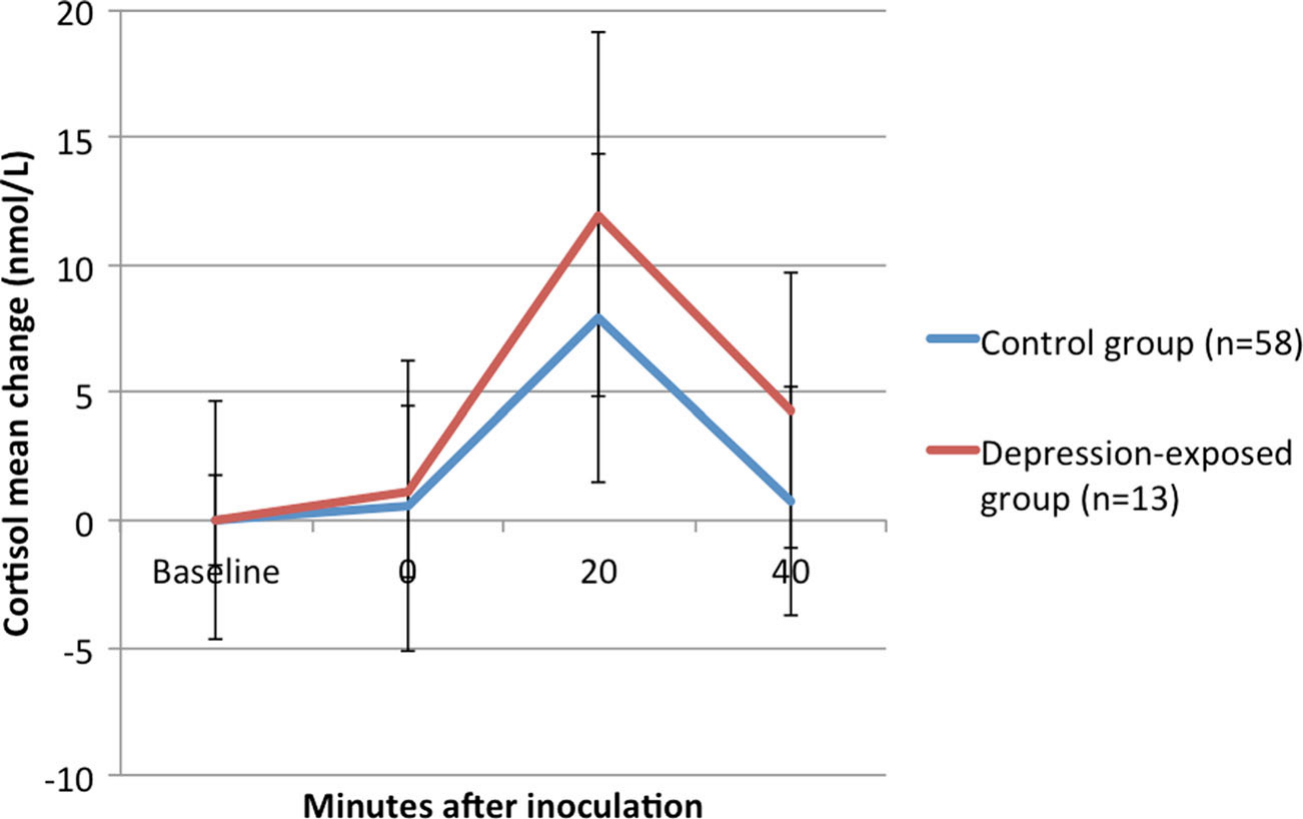
Infant salivary cortisol response to inoculation, split by group

Following the removal of outliers, salivary cortisol measures were available for 71 infants. No measures of infant cortisol were significantly correlated with any infant variables, maternal prenatal mood or maternal salivary cortisol. A repeated measures ANOVA was used to assess infant cortisol responses to inoculation. There was a significant within-subjects effect of time (*F*_(1)_ = 2.949, *p* = 0.035), reflecting an increase in salivary cortisol from baseline to 20 min post-inoculation (*t*_*(*60)_ = −5.250, *p* < 0.001), followed by a decrease in salivary cortisol from 20 to 40 min post-inoculation (*t*_(56)_ = 6.415, *p* < 0.001), see Fig. 2. However, there were no between-subjects effects of group, infant gender, or a gender × group interaction. There was also no effect of maternal postnatal depression on infant cortisol reactivity, but there was a significant effect of infant age (*F*_(1)_ = 7.808, *p* = 0.008). The negative correlation (*r =* −0.260, *p* = 0.043) suggests that younger infants have larger cortisol responses. Maternal cortisol reactivity was not predictive of infant cortisol reactivity; mean cortisol change in response to the infant distress stimulus did not predict infant cortisol reactivity in the regression model (*β* = −0.082, *p* = 0.535). The time of infant feed before inoculation, and whether the feed was breast milk or formula was not associated with the salivary cortisol response.

## Discussion

This was a short-term longitudinal study designed to test for effects of prenatal depressive symptoms on maternal salivary cortisol reactivity in mid and late pregnancy, and on infant cortisol reactivity to inoculation at 2 months of age. Contrary to our initial hypotheses, symptoms of depression were not associated with maternal hyper-cortisol secretion in response to the infant distress stimulus. Further, neither maternal prenatal depressive symptoms nor cortisol reactivity were directly associated with infant cortisol reactivity to inoculation.

Previous work from our group demonstrated that in early pregnancy, participants with symptoms of depression had a significant cortisol response to an infant distress stimulus, whereas a group of non-depressed control participants did not (Murphy et al. 2015). However, the current study failed to reproduce these findings in mid and late pregnancy, despite using the same stressful stimulus. A number of other studies have also failed to report associations between maternal prenatal mood disturbance and increased cortisol levels (Evans et al. 2008; Hellgren et al. 2013; Pluess et al. 2012), and there is evidence to suggest that depression may only be associated with raised cortisol when co-morbid with anxiety (Evans et al. 2008). One plausible explanation for the non-replication is related to the rise in serum cortisol levels as gestation progresses, so that by term serum cortisol concentrations are higher than in the non-pregnancy state, regardless of mood. As such, detecting changes in cortisol in response to an acute stressor in later pregnancy may become difficult, as baseline cortisol concentrations may be very close to ceiling levels. Alternatively, the infant distress video may not have been a sufficiently potent stressor to induce a cortisol stress response in this group of pregnant women, although participants did report increases in state anxiety following the film. Thus, in this cohort, in mid and late pregnancy, there is a clear disparity between reported psychological and biological responses to infant distress.

One potential explanation is that during the course of pregnancy, there is evidence for the development of attentional biases towards distressed compared with non-distressed infant faces (Pearson et al. 2010). Thus, using an infant distress stimulus as an acute stressor may be an inappropriate probe of the HPA axis, given that the full extent and mechanisms by which attentional biases towards infant cues change during pregnancy is currently unclear. Notably, previous studies of stress reactivity in pregnancy have typically used the Trier Social Stress Test (TSST) to successfully probe prenatal HPA function, and have reported significant increases in salivary cortisol in response to this stressor throughout gestation (de Weerth and Buitelaar 2005a; Nierop et al. 2006). Thus, the TSST may be a more reliable probe of HPA function in pregnancy.

The infants in this study showed the expected increase in salivary cortisol in response to inoculation; however, no associations were found between maternal prenatal depressive symptoms and the magnitude of the infant cortisol response. This is in contrast to previous studies that have reported significant associations between maternal prenatal psychological distress and increased neonatal cortisol responses to the heel stick procedure (Davis et al. 2011a; Leung et al. 2010), increased cortisol reactivity to a stressful laboratory task at 3 years of age (de Bruijn et al. 2009) and increased cortisol on the first day of school in 5-year-old children (Gutteling et al. 2005). One possible explanation for the lack of association found here is that the participants were drawn from a low-risk community sample and levels of maternal prenatal depression were relatively low. Had the participants been recruited from a high-risk population with moderate to severe levels of depression, there may have been a significant association between maternal depressive symptoms and infant cortisol reactivity. However, some previous studies have reported associations between maternal prenatal mood disturbance and increased infant cortisol in low-risk community samples (Davis et al. 2011b; Leung et al. 2010), as well as high-risk socio-economically disadvantaged samples (Fernandes et al. 2014), and a clinical sample of depressed participants (Oberlander et al. 2008). Further, contrary to our hypothesis, maternal cortisol reactivity did not directly predict infant cortisol reactivity. A clear limitation, however, is that the infant distress stimulus administered to mothers prenatally failed to induce a salivary cortisol response in the majority of participants. Had a more effective probe of the HPA axis been administered to participants, there may have been an association between maternal and infant cortisol reactivity.

Nonetheless, the lack of convincing evidence of the mediating effects of maternal cortisol in the association between prenatal mood disturbance and adverse offspring outcomes highlights the need to explore alternative mechanisms of effect in this field. Recent evidence has highlighted that epigenetic regulation of gene expression may be an alternative mechanism by which the association between prenatal depression and offspring outcomes is mediated. In particular, there is evidence that maternal prenatal mood may impact upon epigenetic regulation of the gene encoding the glucocorticoid receptor (NR3C1) in offspring. The glucocorticoid receptor plays a critical role in HPA responses to stress through negative feedback on glucocorticoid release, and exposure to prenatal psychological distress has been associated with increased offspring NR3C1 DNA methylation (Braithwaite et al. 2015; Oberlander et al. 2008; Radtke et al. 2011). Further, such epigenetic modifications of this gene have been related to exaggerated cortisol stress responses in infants (Oberlander et al. 2008). Thus, epigenetics may be an important factor when considering the effects of prenatal depression on foetal and infant development, and the existing evidence warrants further investigation.

The short-term longitudinal design and the validated, widely used measure of perinatal depression are significant strengths of this study. However, a number of limitations should be considered. A larger sample size of participants during both the pre- and postnatal phase of the study would have allowed more power to detect differences of small and medium effect sizes. Nonetheless, based on our previously published findings (Murphy et al. 2015), 64 participants would be needed in order to have a 90 % probability of detecting a significant (*p* = 0.05) difference between the two groups. The participants included in this study were not a clinical sample; therefore, the inclusion of solely a clinically depressed sample with more severe depressive symptoms within this cohort would have increased the variability and potentially increased the power to detect group differences. Although we controlled for effects of maternal postnatal depression when testing the association between prenatal depression and infant cortisol reactivity, it is important to note that a number of other postnatal environmental factors could influence infant cortisol reactivity, such as maternal care-giving behaviours. However, such measures were not included in this study, and therefore it was not possible to statistically control for all aspects of the postnatal environment in our analyses. Finally, the stressor administered to the participants failed to induce a significant salivary cortisol response. Although it is unclear whether this was due to the participants being in later gestation at the point of testing, the use of an alternative probe of the HPA axis, such as the TSST, may have yielded a biological stress response.

## Conclusion

To conclude, the most prevalent mechanistic theory in prenatal psychiatry to explain associations between prenatal mood disturbance and adverse offspring outcomes is via alterations of the maternal and infant HPA axes during pregnancy (Braithwaite et al. 2014; Glover 2011; Talge et al. 2007). However, in this study, we found no evidence to suggest that prenatal depression in the second and third trimester is associated with maternal hyper-cortisol reactivity in response to an acute stressor, or infant hyper-cortisol reactivity in response to inoculation. Thus, the role of the HPA axis in mediating these effects may be more subtle and variable than previously thought. Future studies should consider other potential mediating biological mechanisms, such as alterations in maternal sympathetic nervous system activity, changes to maternal and infant immune function and epigenetics, and carefully assess possible timing effects.
